# Dealing with the challenges of the pandemic – results of a population-based survey during the second year of the COVID-19 pandemic contrasting benefits and burden

**DOI:** 10.1186/s12889-024-19203-4

**Published:** 2024-07-19

**Authors:** Alina Geprägs, David Bürgin, Jörg M. Fegert, Elmar Brähler, Vera Clemens

**Affiliations:** 1 Hospital of Child and Adolescent Psychiatry/Psychotherapy, University of Ulm, Ulm, Germany; 2 Child and Adolescent Psychiatric Research Department (UPKKJ), Psychiatric University Hospitals, University of Basel, Basel, Switzerland; 3 Jacobs Center for Productive Youth Development, University of Zurich, Zurich, Switzerland; 4 Department for Psychosomatic Medicine and Psychotherapy, University Medical Center of Johannes, Gutenberg University of Mainz, Mainz, Germany; 5 Integrated Research and Treatment Center Adiposity Diseases, Behavioral Medicine Unit, Department of Psychosomatic Medicine and Psychotherapy, Leipzig University Medical Center, Leipzig, Germany

**Keywords:** Covid-19 pandemic, Family functioning, Quality of life, Parental stress, Mental health problems

## Abstract

**Background:**

The pandemic and the associated consequences have been ongoing stressors with severe impacts on the population and particularly on families. Research focusing on groups dealing well with the challenges of the pandemic is scarce. Here, we aimed to identify groups being well-adjusted during the pandemic and associated predictors.

**Methods:**

A representative sample of the German population (*N* = 2,515, 51.6% women, 50.09 years), and a subsample of persons with children or adolescents under the age of 18 (*N* = 453, 60.3% women, 40.08 years) was assessed from July to October 2021. As huge differences in coping with the pandemic are seen, cluster analysis was performed.

**Results:**

Persons in the “well-adjusted cluster” were characterized by higher quality of life, better coping with the pandemic and lower burden of the pandemic. The family subsample well-adjusted cluster was characterized by lower pandemic-associated burden, lower parental stress compared to before the pandemic and a better relationship with the child. Fewer mental health symptoms and less pandemic-associated negative impact on career predicted membership of the well-adjusted cluster in both samples. An interaction between mental health symptoms and the negative impact of COVID-19 on the career was found.

**Conclusions:**

Our results underscore the importance of mental health and work-related factors for coping with the pandemic.

**Supplementary Information:**

The online version contains supplementary material available at 10.1186/s12889-024-19203-4.

## Introduction

SARS-CoV-2 and the associated respiratory disease COVID-19 first occurred in China in 2019 and subsequentially spread around the world [1]. The cases grew rapidly and forced governments to implement measures to reduce infections, including the closure of childcare institutions, schools and leisure time activities, culture, and gastronomy as well as contact restrictions and curfews.

Although these measures flattened the infection curve and reduced the threat of overloading the resources of the health care systems, they come along with broadly discussed negative consequences for the population including heightened stress and distress [2–4], lower quality of life [5–7], and increased mental health problems [2, 8–10]. In a recent population-based study, we were able to show the importance of mental health for quality of life during the pandemic [11]. Notably, not everyone seemed to be equally affected by the pandemic with certain groups being at increased risk, for example women [6, 8, 12–15], younger people [14–16], people with pre-existing mental and physical health problems [6, 11, 14, 16, 17], people being unemployed [8, 12] and having experienced trauma [14, 15]. Next to risk factors, multiple studies found important protective factors for quality of life and well-being, for example living with a partner [14, 15], older age [6, 8, 12], good financial status [11, 14, 15, 18, 19], doing outdoor activities [8, 11, 12, 14], exercising, resilience and coping skills [15]. Taken together, recent research elucidated important risk and protective factors associated with poor mental health during the pandemic which are unequally distributed across the population.

Together, not everyone has been affected equally and certain groups face specific stressor. In our society, families represent an important subgroup with specific challenges during the pandemic. They were more strongly affected by measures to counteract the pandemic and as such were prone to increased levels of stress and burnout in part due to the loss of daily structures. These changes to family life and functioning included a general heightened uncertainty [20], coordinating working and schooling from home [21], balancing childcare and work duties [22], along with the closure of social activities of children [23]. Parental stress was more commonly reported among women [24, 25], within families having more children [26], co-occurred with younger parental age [27] and pre-existing mental disorders in children [28]. Children and adolescents across all child`s age have been impacted, as reviewed by Cost et al. [29]. Important work-related factors concerning parental stress include pandemic-related changes in working conditions [28], unemployment, lower education, and economic burden [25]. Over and beyond these sociodemographic and work-related factors, parents’ mental health [24, 30, 31], their social support networks [24], along with their own previous stressful life events [34] are important tenets of coping with the pandemic. Although there is research, questioning the higher burden for families, showing no difference to the normal population [35],, considering the high burden on families during and after the pandemic, focusing on healthy family functioning to foster child (mental) health and well-being is necessary and imperative. Taken together, families face specific challenges during the pandemic and therefore should be addressed separately by research.

As resumed above, literature showed several risk factors for the population. But importantly, the pandemic also came along with benefits, such as decreased daily burdens and obligations, a reduction of external stressors and more time for oneself and the core family living in the same household. As most studies focus on risk factors and negative consequences of the pandemic, research about groups of individuals dealing well with the challenges of the pandemic is needed. Notably, about 10% of people experience improvement in work-associated aspects and 13% experience improvements in their private life, associated with more leisure time and care duties, living with a partner and short-time work during the pandemic [36]. Recent data underline the differential effects of the pandemic, with some persons experiencing benefits while others still suffer from long-term effects [37, 38]. Thus, differential reporting based on clustered groups instead of means is needed.

Taken together, literature showed different negative impacts of the pandemic with several risk factors for these negative impacts. Besides the population families faced specific challenges and were more strongly influenced by certain measures. Therefore, families and family functioning should be considered separately. Furthermore, resilience and protective factors have rarely been observed. Within this study, we aimed to identify people who have dealt well with the pandemic within a population-based sample and within a subgroup of participants having children and adolescents via cluster analysis. Furthermore, we aimed to identify risk and protective factors for coping with the pandemic.

## Methods

### Sample procedure

A demographic consulting company (USUMA, Berlin, Germany) obtained the sample. First, a sampling method covering the inhabited area of Germany, called ADM (Arbeitskreis Markt- und Sozialforschungsinstitute e.V.) was used. This is based on the municipal classification of the Federal Republic of Germany. Electronically, Germany is separated into 53,000 areas, each containing around 700 private households. In a second step, these areas are divided into 1,500 regional layers, afterwards into 128 “networks” with 258 single sample points in each. These are proportionate to the distribution of private households in Germany. In a next step, private households were selected systematically, using a random route procedure, where streets were selected randomly. Of these streets, every third residence was invited to participate in the study. With a Kish-selection Grid technique, one person of the household was randomly chosen, if there were more than one fitting person in the household. Speaking German sufficiently and being at least 16 years of age were inclusion criteria. The persons selected were informed about the research background, the research procedure and asked to sign informed consent. Afterwards, an interview was conducted face-to-face at the residence of the participant, asking about basic sociodemographic characteristics. The second part of the survey was completed with a questionnaire filled out by participants themselves with a researcher in the next room to answer possible questions. These two parts were combined in a database without identifying information. The survey was conducted before and at the beginning of the fourth wave of COVID-19 in Germany, between July 28th and October 1st, 2021. At this point of the pandemic, there were no shop, school or childcare facility closures. During the interviews, hygiene measures were implemented (wearing a mask, keeping distance, disinfecting hands). In sum, 5,934 target persons were identified, of which 5,908 were contacted. The most frequent reasons for non-participation were refusal of the selected household to provide information (24.0%), refusal of the target person to participate (13.6%) and failure to contact persons in the household after four attempts (13.4%). All in all, the total sample included *N* = 2,515 participants (utilization rate = 42.6%). For part of the study a subsample (*n* = 453), containing all participants with one or more children or adolescent under 18 was used. Only 3 participants of the whole sample identified as “divers” gender. Therefore, this small group was excluded. The study was conducted in accordance with the Declaration of Helsinki and was approved by the Ethics Committee of the Medical Department of the University of Leipzig.

### Measures

*Life satisfaction* was assessed using a one-item self-rating question („Currently, how satisfied are you all in all with your life?”) with a scale from 0 („not satisfied at all“) to 10 („completely satisfied“) after Beierlein and colleagues [39]. *Coping with the pandemic* was measured using a one-item self-rating question (“All in all, how well did you cope with the challenges of the Corona pandemic?”) with a scale from 0 (“extremely bad”) to 10 (“outstanding”). *Burden of the pandemic* was assessed using a one-item self-rating question (“How much did you feel mentally burdened by the challenges of the Corona pandemic?”) with a scale from 0 (“no mental burden”) to 10 (“extremely strong mental burden”). *Mental health symptoms* were measured using the German version of the Patient Health Questionnaire 4 (PHQ-4) [40, 41]. In our sample, we saw good internal consistency (whole sample: α = 0.86, subsample: α = 0.80). *Own maltreatment experience* was measured using the German version of the ISPCAN Child Abuse Screening Tool – Retrospective (ICAST-R) [42]. For every participant, a sum score with the number of experienced different types of maltreatment was calculated. *Negative career-related impact of COVID-19* was assessed using a one-item self-rating question (“How strongly did the COVID-pandemic affect your career negatively?”) with a scale from 0 (“not at all”) to 10 (“extremely strong”). *Burden of the pandemic for the child* was measured with a one-item self-rating question (“How much is your child mentally burdened by the Corona pandemic?”) with a scale from 0 (“no mental burden”) to 10 (“extremely strong mental burden”). *Change in parental stress* was measured with a modified version of the German Parental Stress Scale [43, 44] with 16 self-rating items referring to changes in parental stress during the pandemic (e.g. “Since the beginning of the pandemic, I`m … with my parent role”) with a scale from − 1 (“more satisfied than before”) to 1 (“less satisfied than before”). A sum score was calculated, with negative values representing negative changes in parenting since the beginning of the pandemic, zero representing no changes and positive values representing positive changes. A good internal consistency (α = 0.94) was seen in our sample. *Relationship with child* was measured with a one-item self-rating question (“Currently, how satisfied are you all in all with the relationship with your child/ your children”) with a scale from 0 (“not satisfied at all”) to 10 (“completely satisfied”). *Division of care duties* was assessed with a one-item self-rating question (“During the pandemic, how satisfied were you with the division of care duties between you and your partner?”) with a scale from 0 (“not at all”) to 10 (“extremely”). *Income* was measured using the equivalent income, which is calculated with the monthly household income and equivalent size of the household which is calculated using giving values for the number of persons living on this income. *Pre-existing somatic disorder/pre-existing psychiatric disorder* were measured with a list of disorders (including cancer, high blood pressure, diabetes, etc. for somatic disorders and Posttraumatic stress disorder (PTSD), Attention Deficit Hyperactivity Disorder (ADHD), eating disorders, etc. for psychiatric disorders) and was coded into a binary variable. With pre-existing disorders was coded as “1”, without preexisting disorders was coded as “0”. *Living alone*, *working from home*, *partner working from home*, i*ncome loss*, and *gender* were coded binary (Living alone (“1”) vs. not living alone (“0”); Working from home (“1”) vs. not working from home (“0”); Partner working from home (“1”) vs. partner not working from home (“0”); Having income loss during the pandemic (“1”) vs. not having had income loss during the pandemic (“0”); female (“0”) vs. male (“1”)). *Depressive symptoms* were assessed with the Patient Health Questionnaire-2 (PHQ2), a screening tool with a sensitivity of 82% and a specificity of 92% for major depressive disorder [45] providing a good internal consistency (ω = 0.77) [46].

### Statistical analysis

All analyses were performed using SPSS version 28. Clustering was performed using a two-step cluster procedure. As distance measure, the log-likelihood was used. As cluster criteria, the bayes criteria (BIC) was used. For the whole sample, life satisfaction, coping with the pandemic and burden of the pandemic were used for clustering, for the subsample burden of the pandemic for the child, change in parental stress and relationship with child were used for clustering. These variables reflect different facets of coping with the pandemic. They were chosen by discussion in the team and based on literature research. As described in the introduction, families faced different challenges than the general population. Therefore, the variables chosen for clustering in the subsample with families are based on family specific aspects. In a next step, comparisons between the clusters were performed with t-tests or Chi^2^-tests. Only variables with significant differences between the clusters were included in the following regression analysis. Afterwards, cluster membership was used as outcome variable in binary logistic regression analysis. For the whole sample, age, income, mental health symptoms, own maltreatment experience, negative impact of COVID-19 on career, gender, pre-existing somatic disorder, pre-existing psychiatric disorder, working from home and income loss were used as independent variables predicting cluster membership. For the subsample, mental health symptoms, own maltreatment experience, division of care duties, pre-existing psychiatric disorder, working from home, partner working from home and income loss were used as independent variables. Income was standardized before this analysis. As mental health symptoms and negative impact of COVID-19 on career appeared to be important predictors for coping with the pandemic, exploratory moderation analyses were performed using the PROCESS Macro [47], to investigate a possible interplay between these two factors. All variables were standardized before the moderation analyses. P-levels are considered as statistically significant at 0.05. Missing data was dealt with listwise deletion.

## Results

As described above, the impact of the pandemic is different for families with children and adolescents compared to the general population. Thus, we performed separate analysis for the general sample and for participants who reported having children and adolescents under the age of 18. The final general sample comprised 2,515 participants, including 1,297 (51.60%) women. Mean age of participants was 50.09 years (SD = 18.05). For an age distribution see Appendix S1. The subsample with children and adolescents for investigating families comprised 453 participants, including 273 (60.3%) women. Mean age of participants was 40.08 years (SD = 8.53). The sample characteristics are presented in Tables 1 and 2. For correlation matrixes for the whole sample and the subsample with families see Appendix S2 and S3.

**Table 1:** Sample characteristics for the whole sample. Presented as mean, standard deviation, range and possible scale range for continuous variables and frequency and percentage for categorical variables. Mental health symptoms were measured with the PHQ-4, own maltreatment experience with the ICAST-R

Variable	*N*	M/*n*	SD/%	Range (scale range)
Age	2515	50.09	18.05	16–101
Equalized household income	2470	2015.05	1012.12	125–7500
Mental health symptoms	2514	1.61	2.15	0–12 (0–12)
Own maltreatment experience	2515	0.70	1.09	0–4 (0–4)
Negative impact of COVID-19 on career	2461	2.36	2.92	0–10 (0–10)
Life satisfaction	2505	7.40	2.09	0–10 (0–10)
Coping with the pandemic	2502	7.15	2.15	0–10 (0–10)
Burden of the pandemic	2502	4.20	3.06	0–10 (0–10)
Gender	2514			
Female		1297	51.60%	-
Pre-existing psychiatric disorder	2456			
Yes		413	16.80%	-
Pre-existing somatic disorder	2502			
Yes		928	37.10%	-
Living alone	2515			
Yes		995	39.60%	-
Working from home	2386			
Yes		467	19.60%	-
Income loss	2471			
Yes		483	19.50%	-

**Table 2:** Sample characteristics for the subsample with children and adolescents. Presented as mean, standard deviation, range and possible scale range for continuous variables and frequency and percentage for categorical variables. Mental health symptoms were measured with the PHQ-4, own maltreatment experience with the ICAST-R, change in parental stress with an adaption of the parental stress scale

Variable	*N*	M/*n*	SD/%	Range
Age	453	40.08	8.53	19–81
Equalized household income	444	1866.48	831.73	125–5303
Mental health symptoms	453	1.39	1.84	0–9 (0–12)
Own maltreatment experience	453	0.69	1.13	0–4 (0–4)
Number of children	440	1.72	0.77	1–5
Age of first child	453	11.41	6.77	0–43
Division of care duties	374	7.08	2.24	0–10 (0–10)
Negative impact of COVID-19 on career	447	2.93	3.08	0–10 (0–10)
Burden of the pandemic for the child	441	4.46	3.21	0–10 (0–10)
Change in parental stress	431	-0.88	3.96	-14-10 (-16-16)
Relationship with child	439	8.81	1.48	1–10 (0–10)
Gender	453			
Female		273	60.30%	-
Pre-existing psychiatric disorder	444			
Yes		63	14.20%	-
Pre-existing somatic disorder	450			
yes		88	19.60%	-
Working from home	447			
Yes		107	23.90%	-
Partner working from home	356			
Yes		87	24.40%	
Income loss	444			
Yes		122	27.50%	-

### Cluster analysis

**Fig. 1:**
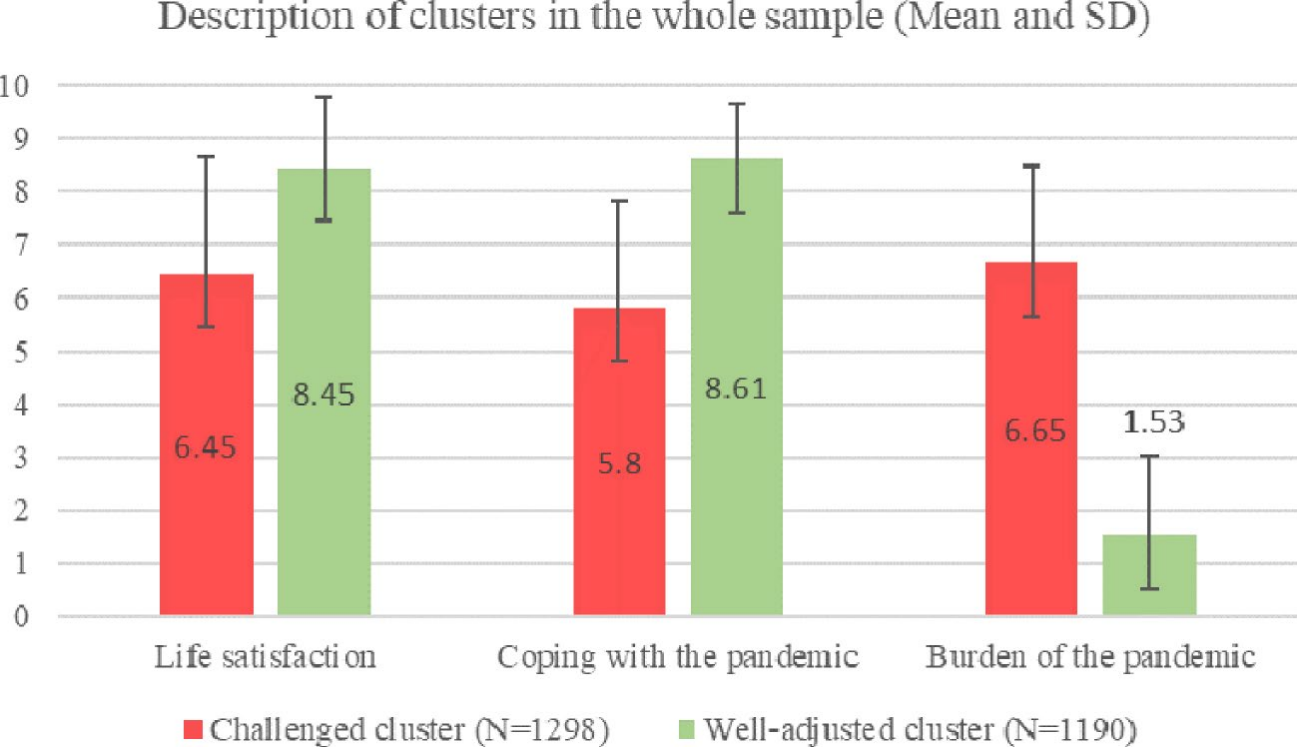
Description of clusters in the whole sample using the variables used for clustering. Mean and SD are displayed; Range: 1–10. Higher values represent higher quality of life, better coping with the pandemic and higher burden of the pandemic

The cluster analysis to identify people who dealt well with the pandemic-associated changes in the general sample resulted in two clusters with satisfying quality (see Appendix S4). One cluster is characterized by higher life satisfaction, better coping with the pandemic and lower burden due to the pandemic, therefore being the well-adjusted cluster. The other cluster is characterized by lower life satisfaction, worse coping with the pandemic and higher burden due to the pandemic, therefore being the challenged cluster. A description of the clusters is shown in Fig. 1.

**Fig. 2:**
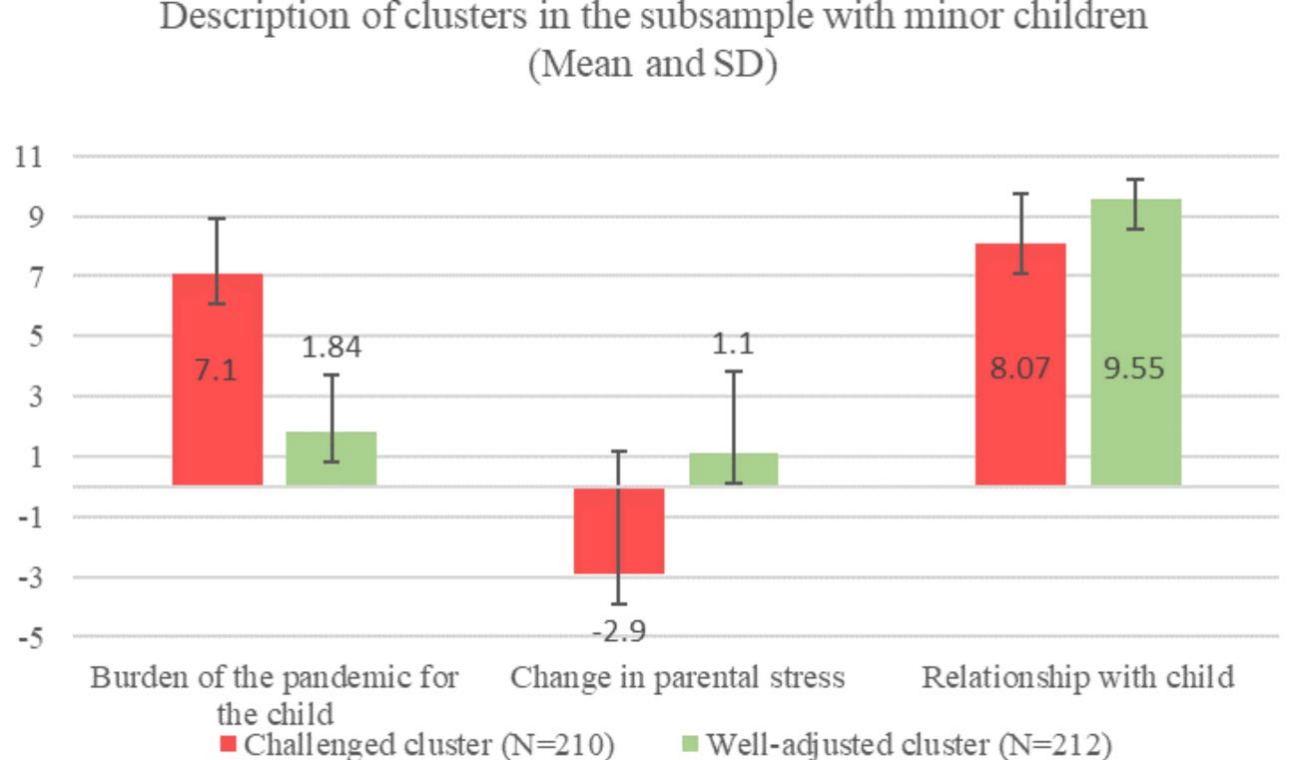
Description of clusters in the subsample with children and adolescents for the variables used for clustering Mean and SD are displayed; Range: 1–10. Higher values represent higher burden for the child, more bettering in parental stress and better relationship with child

The cluster analysis identifying parents who dealt well with pandemic-associated changes in the subsample of families with children and adolescents resulted in two clusters with satisfying quality (see Appendix S5). One cluster is characterized by lower pandemic-associated burden, positive change in parental stress and better relationship with child, therefore named the well-adjusted cluster. The other cluster is characterized by higher burden, negative change in parental stress and worse relationship with child, therefore named the challenged cluster. A description of the clusters is shown in Fig. 2.

### Factors associated with cluster membership

To identify factors contributing to belonging to a cluster, binary regression analysis with “cluster membership” as dependent variable were performed. In a first analysis, differences between the clusters were checked, using t-tests and Chi-square Tests (see Appendix S6 and S7). Only variables with significant differences in the previous analysis were included as predictors in the main regression analysis. Focusing on the general sample, age, equalized household income, mental health symptoms, own maltreatment experience, negative impact of COVID-19 on career, gender, pre-existing somatic disorder, pre-existing psychiatric disorder, working from home and income loss were used as predictors (see Table 3). Income was standardized before the analysis. Younger age, higher income, fewer mental health symptoms, fewer own maltreatment experiences, less negative career-related impacts of COVID-19 and no pre-existing psychiatric disorders increased the odds of belonging to the “well-adjusted cluster”. The results are displayed in Table 3.

**Table 3:** Associations of core variables with cluster membership using the whole sample. Presented as odds ratio (OR). An OR > 1 corresponds to a higher probability of belonging to the well-adjusted cluster with increasing values of the predictor. p-values < 0.001 are marked with ***, p-values < 0.01 are marked with ** and p-values < 0.05 are marked with *. Cox & Snell R^*2*^ *= 0.30;N = 2,191*

Predictor	OR	OR 95%CI	*p*
Constant	8.11***	-	< 0.001
Age	0.99***	0.98–0.99	< 0.001
Equalized household income	1.14*	1.02–1.27	0.02
Mental health symptoms	0.71***	0.66–0.76	< 0.001
Own maltreatment experience	0.82***	0.74–0.91	< 0.001
Negative impact of COVID-19 on career	0.69***	0.66–0.73	< 0.001
Male gender	1.13	0.92–1.38	0.26
Pre-existing somatic disorder	0.88	0.69–1.13	0.32
Pre-existing psychiatric disorder	0.52***	0.37–0.72	< 0.001
Working from home	0.84	0.64–1.10	0.19
Income loss	0.96	0.72–1.29	0.80

Focusing on the subsample of participants with children and adolescents, mental health symptoms, own maltreatment experience, division of care duties, negative impact of COVID-19 on career, pre-existing psychiatric disorder, working from home, and income loss were used as predictors. A higher probability of being in the well-adjusted cluster was associated with higher satisfaction regarding the division of care duties, fewer mental health symptoms and fewer negative career-related impacts of the COVID pandemic. Being a member of the well-adjusted cluster was predicted by higher satisfaction regarding the division of care duties, fewer mental health symptoms, and fewer negative career-related impacts of the COVID pandemic. The results are displayed in table 4

**Table 4:** Associations of core variables with cluster membership using the subsample withchildren and adolescents. Presented as odds ratio (OR). An OR > 1 corresponds to a higher probability of belonging to the well-adjusted cluster with increasing values of the predictor. p-values < 0.001 are marked with ***, p-values < 0.01 are marked with ** and p-values < 0.05 are marked with *. Cox & Snell R^*2*^ *= 0.24;N = 331*

Predictor	OR	OR 95%CI	*p*
Constant	1.47	-	0.45
Mental health symptoms	0.79*	0.66–0.95	0.01
Own maltreatment experience	0.78	0.59–1.03	0.08
Division of care duties	1.16*	1.03–1.30	0.02
Negative impact of COVID-19 on career	0.79***	0.72–0.87	< 0.001
Pre-existing psychiatric disorder	0.81	0.33–2.01	0.65
Working from home	0.75	0.42–1.37	0.35
Income loss	0.83	0.45–1.54	0.56

### Moderation analysis

As mental health symptoms and negative impact of COVID-19 on career appeared to be important predictors for coping with the pandemic, exploratory moderation analysis was performed to better understand the impact of pandemic-associated changes in the career on the association between mental health and coping with the pandemic. Cluster membership served as dependent variable, mental health symptoms as independent variable and negative impact of COVID-19 on career as moderator. We found a significant interaction between mental health and pandemic-associated impairments in the career which predicted belonging to the well-adjusted cluster. The results are displayed in Table 5. With higher negative impact of COVID-19 on career the effect of mental health symptoms is less strong. For a graphic representation see Appendix S8.

**Table 5:** Moderation analysis using the whole sample. Presented as odds ratio (OR). An OR > 1 corresponds to a higher probability of belonging to the well-adjusted cluster with increasing values of the predictor. p-values < 0.001 are marked with ***, p-values < 0.01 are marked with ** and p-values < 0.05 are marked with *. Cox & Snell R^*2*^ *= 0.28;N = 2,442*

Predictor	OR	OR 95%CI	*p*
Constant	0.79***	0.71–0.87	< 0.001
Mental health symptoms (independent variable)	0.38***	0.34–0.44	< 0.001
Negative impact of COVID-19 on career (moderator)	0.38***	0.34–0.43	< 0.001
Interaction term	1.32***	1.15–1.51	< 0.001

In the subsample of parents with children and adolescents, a significant interaction was seen. With higher negative impact of COVID-19 on career the effect of mental health symptoms is less strong. The results are displayed in table 6. For a graphic representation see appendix S9

**Table 6:** Moderation analysis for the family cluster. Presented as odds ratio (OR). An OR > 1 corresponds to a higher probability of belonging to the well-adjusted cluster with increasing values of the predictor. p-values < 0.001 are marked with ***, p-values < 0.01 are marked with ** and p-values < 0.05 are marked with *. Cox & Snell R^*2*^ *= 0.23;N = 422*

Predictor	OR	OR 95%CI	*p*
Constant	0.92	0.74–1.16	0.50
Mental health symptoms (independent variable)	0.46***	0.36–0.61	< 0.001
Negative impact of COVID-19 on career (moderator)	0.45***	0.36–0.58	< 0.001
Interaction term	1.35**	1.05–1.75	0.02

## Discussion

To the best of our knowledge, this is the first study in a large population-based sample in Germany identifying persons dealing well with the pandemic, focusing on both, benefits and burden in the general population and families with children and adolescents.

Results reveal that fewer current mental health symptoms predicted membership of the well-adjusted cluster in both samples. This is in line with previous research, showing associations between mental health symptoms and reduced quality of life before and during the pandemic [46–48]. Thus, our result underscores the importance of mental health for dealing well with crisis, possibly due to a lack of coping strategies, which was shown for people with psychiatric disorders [48]. Furthermore, pre-existing psychiatric disorders predicted belonging to the cluster of persons challenged by the pandemic in the total sample. In the family sample, we did not find any significant association between belonging to the well-adjusted cluster and pre-existing psychiatric disorders. This could be due to the reduced size of the subsample and the included current mental health symptoms drawing too much variance.

Having experienced fewer forms of maltreatment in one´s own childhood predicted membership of the well-adjusted cluster for the whole sample. This is in line with research showing that people with maltreatment experience in childhood have more negative coping mechanisms [49]: This is a risk, especially in times of crisis when positive coping strategies are needed to deal with heightened stress. Interestingly, own maltreatment experience was no significant predictor for membership of the well-adjusted cluster in the family subsample. This is surprising, considering the association between own maltreatment experience and parental stress, as we recently confirmed in another study [50]. Nevertheless, this result should be examined further in future research, regarding the potential negative consequences of being in the challenged cluster. For the families, parental stress and parent-child relationship were used. Both have been showed to be associated to harsh parenting [27, 51] and therefore to be important in child protection prevention. On the other hand, our result could suggest that not the experience of own maltreatment alone heightens the risk of poor coping, but subsequent psychiatric diseases, which develop after the maltreatment experience. Therefore this result may again confirm the importance of mental health symptoms. Finally, similarly to pre-existing psychiatric disorders, the small sample size and possible other related variables, such as mental health symptoms and parental stress, may play a role here.

Focusing on sociodemographic variables, younger age and higher income predicted membership of the well-adjusted clusters. This is in line with some research, showing associations between financial factors and quality of life and mental health during the pandemic [11, 15, 18]. On the other hand, this contrasts findings that younger age is associated with a higher mental health burden during the pandemic [14–16]. One explanation could be that along the course of the pandemic, younger people adapt better to the situation. During the time of this survey, there was no severe lockdown in Germany, therefore younger people were probably influenced to a smaller degree during this point of time. Furthermore, gender was no significant predictor for cluster membership, contrasting studies showing associations between female gender and lower quality of life and higher mental health burden during the pandemic [12, 15, 18]. Again, the later stage of the pandemic may play a role here. Moreover, in our study, we focused not only on quality of life but on different factors influencing our cluster analysis, potentially equaling gender differences. Somatic disorders were no significant predictor for cluster membership. This too, could be due to the less restrictive measures, as the health care system wasn`t as much overloaded at this time in Germany.

Concerning pandemic-related factors, the negative impact of COVID-19 on the career predicted membership of the challenged cluster, while working from home and income loss were no significant predictors, contrary to previous studies, showing associations between income loss during the pandemic and worsened mental health [18]. This may point towards the importance of one`s own perception of work-related impacts, focusing on subjective perception in contrast to income loss and working from home.

Taken together for the general population fewer mental health symptoms, no negative impact on career, younger age, higher income, and fewer own maltreatment experiences predicted better coping with the pandemic. Therefore, these resilience factors being a starting point to increase resilience of the population by addressing them with measures. Furthermore, underlining the importance of mental health support to increase resilience and preventing long-term negative consequences due to crisis.

Focusing on participants with children and adolescents, the relevance of satisfaction with the division of care duties was highlighted. Being more satisfied with this division predicted membership of the well-adjusted cluster. Taking into account the higher burden and adverse outcomes that have been shown for caregivers during the pandemic [54], this is not surprising. Previous research has already shown the relevance of satisfaction with childcare duties for harmful parenting behavior during the pandemic [55], showing the importance for parents supporting each other in care. Yet, future research could examine the age spectrum of children and adolescents divided into smaller age groups, to adequately address the different challenges for different age groups. In a review older age of the child has been shown to be a risk factor for poor mental health, with the authors concluding the contact restrictions having greater impact on adolescents because of their greater peer orientation [56]. Furthermore, excessive media consumption [56] and risky behaviour like substance abuse being adolescent-specific challenges during the pandemic [57].

Concerning the interplay between work-related factors and mental health, our exploratory moderation analysis revealed an interaction between mental health burden and negative impact of COVID-19 on career in both clusters. In detail, with increased negative impact of COVID-19 on the career the effect of mental health symptoms is weaker. Associations between higher job strain and job insecurity with poor mental health have been frequently shown in literature [58, 59]. Besides, being unemployed seems to be worse for mental health [60]. Transferring this to the pandemic, a worsening in working conditions and career could be a threat to mental health and coping with the pandemic. Our results elucidate this interplay. Taken together, this hints towards the importance of both mental health and work-related factors for dealing well with the challenges of the pandemic. Practically, to provide targeted support, work-related factors as well as mental health burden should be assessed. Therefore, psychotherapeutic and social work support need to be implemented together, to install effective support. All in all, this finding is of particular importance for the effectiveness of individual targeted support. People may need both, work-related support and mental health support to increase the succession rate of the treatments.

### Strengths and limitations

The main strength of our study is the large-scaled, population-based sample, which is representative for the German population. Besides, we investigated various sociodemographic, psychological and pandemic-related variables. However, there are some limitations to consider. Only short self-reporting measures were used, due to the many different factors which were investigated to reduce the overall time effort for the participants. Longer questionnaires for specific factors could therefore gain further insights into these specific factors. These self-reporting measures could be influenced by social desirability, even though the anonymous implementation of the questionnaire withstands this limitation to some degree. Our study has a cross-sectional design. Nothing can be said about the timing of events and causality. Therefore, we cannot exclude the possibility of confounding factors, influencing our results and the changes being arisen only by the pandemic. Longitudinal research during different phases of the pandemic would enable us to gain insights into changes over time and therefore identify risk groups for long-term negative consequences. Besides these limitations, our study provides important insights into resilient groups during the pandemic, the characteristics of these groups and the interplay between work and mental health factors, using a large population-representative sample in Germany.

## Conclusion

Fewer mental health symptoms and less negative impact of COVID-19 on career during the pandemic predicted better coping with the pandemic in the general population of Germany and in participants with children and adolescents. Therefore, low-threshold mental health support is of particular importance for dealing with times of crisis such as the pandemic. In the general population, younger age, higher income, and fewer own maltreatment experiences predicted better coping with the pandemic- In participants with children and adolescents, satisfaction with the division of care duties was a strong predictor for better coping with the pandemic. This underlines the relevance of targeted support for parents in child care. Mental health symptoms and negative impact of COVID-19 on career interacted in the way that the effect of mental health symptoms was smaller when the pandemic-associated impact on the career was negative. This is important, concerning the effectiveness of mental health support, which might be diminished when the negative impacts of COVID-19 on the career were high.

### Electronic supplementary material

Below is the link to the electronic supplementary material.

Supplementary Material 1

## Data Availability

No datasets were generated or analysed during the current study.

## References

[1] RKI. Epidemiologisches Bulletin 12/2020. https://www.rki.de/DE/Content/Infekt/EpidBull/Archiv/2020/Ausgaben/12_20.pdf?__blob=publicationFile; 2020.

[2] Bäuerle A, Steinbach J, Schweda A, Beckord J, Hetkamp M, Weismüller B, et al. Mental Health Burden of the COVID-19 outbreak in Germany: predictors of Mental Health Impairment. J Prim Care Community Health. 2020;11:2150132720953682. 32865107 10.1177/2150132720953682 PMC7457643

[3] Taylor BK, Frenzel MR, Johnson HJ, Willett MP, White SF, Badura-Brack AS, et al. Increases in Stressors Prior to-Versus during the COVID-19 pandemic in the United States are Associated with Depression among Middle-aged mothers. Front Psychol. 2021;12:706120. 34305763 10.3389/fpsyg.2021.706120 PMC8292718

[4] Calvano C, Engelke L, Di Bella J, Kindermann J, Renneberg B, Winter SM. Families in the COVID-19 pandemic: parental stress, parent mental health and the occurrence of adverse childhood experiences-results of a representative survey in Germany. Eur Child Adolesc Psychiatry. 2021:1–13. 10.1007/s00787-021-01739-0 PMC7917379 33646416

[5] Ammar A, Trabelsi K, Brach M, Chtourou H, Boukhris O, Masmoudi L, et al. Effects of home confinement on mental health and lifestyle behaviours during the COVID-19 outbreak: insights from the ECLB-COVID19 multicentre study. Biol Sport. 2021;38(1):9–21. 33795912 10.5114/biolsport.2020.96857 PMC7996377

[6] Ferreira LN, Pereira LN, da Fé Brás M, Ilchuk K. Quality of life under the COVID-19 quarantine. Qual Life Res. 2021;30(5):1389–405. 33389523 10.1007/s11136-020-02724-x PMC7778495

[7] Ravens-Sieberer U, Kaman A, Erhart M, Devine J, Schlack R, Otto C. Impact of the COVID-19 pandemic on quality of life and mental health in children and adolescents in Germany. Eur Child Adolesc Psychiatry. 2021:1–11. 10.1007/s00787-021-01726-5 PMC7829493 33492480

[8] Benke C, Autenrieth LK, Asselmann E, Pané-Farré CA. Lockdown, quarantine measures, and social distancing: associations with depression, anxiety and distress at the beginning of the COVID-19 pandemic among adults from Germany. Psychiatry Res. 2020;293:113462. 32987222 10.1016/j.psychres.2020.113462 PMC7500345

[9] Bendau A, Plag J, Kunas S, Wyka S, Ströhle A, Petzold MB. Longitudinal changes in anxiety and psychological distress, and associated risk and protective factors during the first three months of the COVID-19 pandemic in Germany. Brain Behav. 2021;11(2):e01964. 33230969 10.1002/brb3.1964 PMC7744907

[10] Nelson BW, Pettitt A, Flannery JE, Allen NB. Rapid assessment of psychological and epidemiological correlates of COVID-19 concern, financial strain, and health-related behavior change in a large online sample. PLoS ONE. 2020;15(11):e0241990. 33175882 10.1371/journal.pone.0241990 PMC7657530

[11] Geprägs A, Bürgin D, Fegert JM, Brähler E, Clemens V. The Impact of Mental Health and Sociodemographic Characteristics on Quality of Life and Life Satisfaction during the Second Year of the COVID-19 Pandemic& Results of a Population-Based Survey in Germany. International Journal of Environmental Research and Public Health. 2022;19(14):8734. 35886588 10.3390/ijerph19148734 PMC9316196

[12] Teotônio I, Hecht M, Castro LC, Gandolfi L, Pratesi R, Nakano EY, et al. Repercussion of COVID-19 pandemic on brazilians’ quality of life: a nationwide cross-sectional study. Int J Environ Res Public Health. 2020;17:22. 10.3390/ijerph17228554 PMC7698925 33218087

[13] Abreu L, Koebach A, Díaz O, Carleial S, Hoeffler A, Stojetz W, et al. Life with Corona: increased gender differences in aggression and depression symptoms due to the COVID-19 pandemic Burden in Germany. Front Psychol. 2021;12:689396. 34385959 10.3389/fpsyg.2021.689396 PMC8353131

[14] Ellwardt L, Präg P. Heterogeneous mental health development during the COVID-19 pandemic in the United Kingdom. Sci Rep. 2021;11(1):15958. 34354201 10.1038/s41598-021-95490-w PMC8342469

[15] Fernández RS, Crivelli L, Guimet NM, Allegri RF, Pedreira ME. Psychological distress associated with COVID-19 quarantine: latent profile analysis, outcome prediction and mediation analysis. J Affect Disord. 2020;277:75–84. 32799107 10.1016/j.jad.2020.07.133 PMC7413121

[16] Lingelbach K, Piechnik D, Gado S, Janssen D, Eichler M, Hentschel L, et al. Effects of the COVID-19 pandemic on Psychological Well-Being and Mental Health based on a German online survey. Front Public Health. 2021;9:655083. 34307274 10.3389/fpubh.2021.655083 PMC8296300

[17] Dawel A, Shou Y, Smithson M, Cherbuin N, Banfield M, Calear AL, et al. The Effect of COVID-19 on Mental Health and Wellbeing in a Representative Sample of Australian adults. Front Psychiatry. 2020;11:579985. 33132940 10.3389/fpsyt.2020.579985 PMC7573356

[18] Feter N, Caputo EL, Doring IR, Leite JS, Cassuriaga J, Reichert FF, et al. Sharp increase in depression and anxiety among Brazilian adults during the COVID-19 pandemic: findings from the PAMPA cohort. Public Health. 2021;190:101–7. 33387848 10.1016/j.puhe.2020.11.013 PMC7773543

[19] Gadermann AC, Thomson KC, Richardson CG, Gagné M, McAuliffe C, Hirani S, et al. Examining the impacts of the COVID-19 pandemic on family mental health in Canada: findings from a national cross-sectional study. BMJ Open. 2021;11(1):e042871. 33436472 10.1136/bmjopen-2020-042871 PMC7804831

[20] Achterberg M, Dobbelaar S, Boer OD, Crone EA. Perceived stress as mediator for longitudinal effects of the COVID-19 lockdown on wellbeing of parents and children. Sci Rep. 2021;11(1):2971. 33536464 10.1038/s41598-021-81720-8 PMC7859207

[21] Alonzi S, Park JE, Pagán A, Saulsman C, Silverstein MW. An examination of COVID-19-Related stressors among parents. Eur J Investig Health Psychol Educ. 2021;11(3):838–48. 34563074 10.3390/ejihpe11030061 PMC8544229

[22] Carroll N, Sadowski A, Laila A, Hruska V, Nixon M, Ma DWL et al. The impact of COVID-19 on Health Behavior, stress, Financial and Food Security among Middle to High Income Canadian families with Young Children. Nutrients. 2020;12(8). 10.3390/nu12082352 PMC7468859 32784530

[23] Mohler-Kuo M, Dzemaili S, Foster S, Werlen L, Walitza S. Stress and Mental Health among Children/Adolescents, their parents, and young adults during the First COVID-19 Lockdown in Switzerland. Int J Environ Res Public Health. 2021;18(9). 10.3390/ijerph18094668 PMC8124779 33925743

[24] Johnson MS, Skjerdingstad N, Ebrahimi OV, Hoffart A, Johnson SU. Parenting in a pandemic: parental stress, anxiety and depression among parents during the government-initiated physical distancing measures following the first wave of COVID-19. Stress Health. 2021. 10.1002/smi.3120 34902219

[25] Park H, Choi S, Noh K, Hong JY. Racial discrimination as a cumulative risk factor affecting parental stress on the psychological distress of Korean americans (both US- and Foreign-Born) amid COVID-19: structural equation modeling. J Racial Ethn Health Disparities. 2021:1–10. 10.1007/s40615-021-01106-4 PMC8288412 34282523

[26] Avery AR, Tsang S, Seto EYW, Duncan GE. Differences in stress and anxiety among women with and without children in the Household during the early months of the COVID-19 pandemic. Front Public Health. 2021;9:688462. 34540782 10.3389/fpubh.2021.688462 PMC8440851

[27] Marzilli E, Cerniglia L, Tambelli R, Trombini E, De Pascalis L, Babore A, et al. The COVID-19 pandemic and its impact on families’ Mental Health: the Role played by parenting stress, parents’ past trauma, and Resilience. Int J Environ Res Public Health. 2021;18:21. 10.3390/ijerph182111450 PMC8583183 34769967

[28] Chung G, Lanier P, Wong PYJ. Mediating effects of parental stress on harsh parenting and parent-child relationship during coronavirus (COVID-19) pandemic in Singapore. J Family Violence. 2020. 10.1007/s10896-020-00200-1 PMC7467635 32895601

[29] Cusinato M, Iannattone S, Spoto A, Poli M, Moretti C, Gatta M, et al. Stress, resilience, and well-being in Italian children and their parents during the COVID-19 pandemic. Int J Environ Res Public Health. 2020;17:22. 10.3390/ijerph17228297 PMC7696524 33182661

[30] Cost KT, Crosbie J, Anagnostou E, Birken CS, Charach A, Monga S, et al. Mostly worse, occasionally better: impact of COVID-19 pandemic on the mental health of Canadian children and adolescents. Eur Child Adolesc Psychiatry. 2022;31(4):671–84. 33638005 10.1007/s00787-021-01744-3 PMC7909377

[31] Wu M, Xu W, Yao Y, Zhang L, Guo L, Fan J, et al. Mental health status of students’ parents during COVID-19 pandemic and its influence factors. Gen Psychiatr. 2020;33(4):e100250. 34192232 10.1136/gpsych-2020-100250 PMC7387315

[32] Brown SM, Doom JR, Lechuga-Peña S, Watamura SE, Koppels T. Stress and parenting during the global COVID-19 pandemic. Child Abuse Negl. 2020:104699. 10.1016/j.chiabu.2020.104699 PMC7440155 32859394

[33] Farah R, Zivan M, Niv L, Havron N, Hutton J, Horowitz-Kraus T. High screen use by children aged 12–36 months during the first COVID-19 lockdown was associated with parental stress and screen use. Acta Paediatr. 2021;110(10):2808–9. 34110031 10.1111/apa.15979

[34] Moscardino U, Dicataldo R, Roch M, Carbone M, Mammarella IC. Parental stress during COVID-19: a brief report on the role of distance education and family resources in an Italian sample. Curr Psychol. 2021;40(11):5749–52. 33613013 10.1007/s12144-021-01454-8 PMC7882229

[35] Ecker A, Jarvers I, Schleicher D, Kandsperger S, Schelhorn I, Meyer M, et al. Problems or prospects? Being a parent in the early phase of the COVID-19 pandemic in Germany. Front Psychol. 2022;13:901249. 35992448 10.3389/fpsyg.2022.901249 PMC9389411

[36] Tušl M, Brauchli R, Kerksieck P, Bauer GF. Impact of the COVID-19 crisis on work and private life, mental well-being and self-rated health in German and Swiss employees: a cross-sectional online survey. BMC Public Health. 2021;21(1):741. 33865354 10.1186/s12889-021-10788-8 PMC8052554

[37] Ravens-Sieberer U, Devine J, Napp A-K, Kaman A, Saftig L, Gilbert M et al. Three years into the pandemic: results of the longitudinal German COPSY study on youth mental health and health-related quality of life. Front Public Health. 2023;11. 10.3389/fpubh.2023.1129073 PMC10307958 37397777

[38] Cosma A, Bersia M, Abdrakhmanova S, Badura P, Gobina I. Coping through crisis: COVID-19 pandemic experiences and adolescent mental health and well-being in the WHO European Region: impact of the COVID-19 pandemic on young people’s health and well-being from the findings of the HBSC survey round 2021/2022. Copenhagen: World Health Organization. Regional Office for Europe; 2023 2023. Contract No.: WHO/EURO:2023-7680-47447-69735.

[39] Beierlein C, Kovaleva A, László Z, Kemper CJ, Rammstedt B. Kurzskala zur Erfassung der Allgemeinen Lebenszufriedenheit (L-1). Zusammenstellung sozialwissenschaftlicher Items und Skalen. https://www.gesis.org/kurzskalen-psychologischer-merkmale/download/2015.2015 [.

[40] Kroenke K, Spitzer RL, Williams JB, Löwe B. An ultra-brief screening scale for anxiety and depression: the PHQ-4. Psychosomatics. 2009;50(6):613–21. 19996233 10.1176/appi.psy.50.6.613

[41] Löwe B, Wahl I, Rose M, Spitzer C, Glaesmer H, Wingenfeld K, et al. A 4-item measure of depression and anxiety: validation and standardization of the Patient Health Questionnaire-4 (PHQ-4) in the general population. J Affect Disord. 2010;122(1–2):86–95. 19616305 10.1016/j.jad.2009.06.019

[42] Dunne MP, Zolotor AJ, Runyan DK, Andreva-Miller I, Choo WY, Dunne SK, et al. ISPCAN child abuse screening tools retrospective version (ICAST-R): Delphi study and field testing in seven countries. Child Abuse Negl. 2009;33(11):815–25. 19853301 10.1016/j.chiabu.2009.09.005

[43] Stadelmann S, Perren S, Kölch M, Groeben M, Schmid M. Psychisch kranke und unbelastete Eltern. Kindh Entwickl. 2010;19(2):72–81. 10.1026/0942-5403/a000011

[44] Berry JO, Jones WH. The parental stress scale: initial psychometric evidence. J Social Personal Relationships. 1995;12(3):463–72. 10.1177/0265407595123009

[45] Löwe B, Kroenke K, Gräfe K. Detecting and monitoring depression with a two-item questionnaire (PHQ-2). J Psychosom Res. 2005;58(2):163–71. 15820844 10.1016/j.jpsychores.2004.09.006

[46] Wicke FS, Krakau L, Löwe B, Beutel ME, Brähler E. Update of the standardization of the Patient Health Questionnaire-4 (PHQ-4) in the general population. J Affect Disord. 2022;312:310–4. 35760191 10.1016/j.jad.2022.06.054

[47] Hayes AF. In: Little TD, editor. Introduction to Mediation, Moderation, and conditional process analysis a regression-based Approach. New York: Guilford Publications, Inc.; 2017.

[48] Öztürk Çopur E, Karasu F. The impact of the COVID-19 pandemic on the quality of life and depression, anxiety, and stress levels of individuals above the age of eighteen. Perspect Psychiatr Care. 2021;57(4):1645–55. 33512758 10.1111/ppc.12730 PMC8014617

[49] Hohls JK, König HH, Quirke E, Hajek A, Anxiety. Depression and Quality of Life-A systematic review of evidence from Longitudinal Observational studies. Int J Environ Res Public Health. 2021;18(22). 10.3390/ijerph182212022 PMC8621394 34831779

[50] Huber MB, Felix J, Vogelmann M, Leidl R. Health-Related Quality of Life of the General German Population in 2015: results from the EQ-5D-5L. Int J Environ Res Public Health. 2017;14(4). 10.3390/ijerph14040426 PMC5409627 28420153

[51] Milojevich HM, Levine LJ, Cathcart EJ, Quas JA. The role of maltreatment in the development of coping strategies. J Appl Dev Psychol. 2018;54:23–32. 32489226 10.1016/j.appdev.2017.10.005 PMC7266099

[52] Geprägs A, Bürgin D, Fegert JM, Brähler E, Clemens V. Parental stress and physical violence against children during the second year of the COVID-19 pandemic: results of a population-based survey in Germany. Child Adolesc Psychiatry Ment Health. 2023;17(1):25. 36804027 10.1186/s13034-023-00571-5 PMC9940081

[53] Stith SM, Liu T, Davies LC, Boykin EL, Alder MC, Harris JM, et al. Risk factors in child maltreatment: a meta-analytic review of the literature. Aggress Violent Beh. 2009;14(1):13–29. 10.1016/j.avb.2006.03.006

[54] Beach SR, Schulz R, Donovan H, Rosland AM. Family Caregiving during the COVID-19 pandemic. Gerontologist. 2021;61(5):650–60. 33847355 10.1093/geront/gnab049 PMC8083337

[55] Clemens V, Köhler-Dauner F, Ziegenhain U, Fegert JM. Predictors of parental coping during the Covid-19 pandemic: a Survey in Germany. Front Psychol. 2021;12:715327. 34566797 10.3389/fpsyg.2021.715327 PMC8460925

[56] Panchal U, Salazar de Pablo G, Franco M, Moreno C, Parellada M, Arango C, et al. The impact of COVID-19 lockdown on child and adolescent mental health: systematic review. Eur Child Adolesc Psychiatry. 2023;32(7):1151–77. 34406494 10.1007/s00787-021-01856-w PMC8371430

[57] Meherali S, Punjani N, Louie-Poon S, Abdul Rahim K, Das JK, Salam RA et al. Mental Health of children and adolescents amidst COVID-19 and Past Pandemics: a Rapid systematic review. Int J Environ Res Public Health. 2021;18(7). 10.3390/ijerph18073432 PMC8038056 33810225

[58] Stansfeld S, Candy B. Psychosocial work environment and mental health–a meta-analytic review. Scand J Work Environ Health. 2006;32(6):443–62. 17173201 10.5271/sjweh.1050

[59] Cottini E, Lucifora C. Mental Health and Working conditions in Europe. ILR Rev. 2013;66(4):958–88. 10.1177/001979391306600409

[60] Llena-Nozal A. The Effect of Work Status and Working conditions on Mental Health in four OECD Countries. Natl Inst Econ Rev. 2009;209:72–87. 10.1177/0027950109345234

