# Supplementary material for: Dealing with the challenges of the pandemic – results of a population-based survey during the second year of the COVID-19 pandemic contrasting benefits and burden
Source: BMC Public Health. 2024 Jul 19;24:1941. doi: 10.1186/s12889-024-19203-4 (PMC11264980; doi:10.1186/s12889-024-19203-4)
Supplement: Supplementary file 1 — Supplementary Material 1 [file 12889_2024_19203_MOESM1_ESM.docx]

**Supplemental material - Dealing with the challenges of the pandemic – results of a population based survey during the second year of Covid 19 pandemic contrasting benefits and burden**

**Appendix 1**

| **Age category in years** | **N** | **%** |
| --- | --- | --- |
| 16-19 | 98 | 3.9 |
| 20-29 | 331 | 13.2 |
| 30-39 | 354 | 14.1 |
| 40-49 | 395 | 15.7 |
| 50-59 | 510 | 20.3 |
| 60-69 | 442 | 17.6 |
| 70-79 | 257 | 10.2 |
| 80-89 | 122 | 4.9 |
| 90-101 | 6 | 0.2 |

**Appendix 2**

*Correlation matrix for the whole sample.*

| **Variable** | **Age** | **Income** | **Mental health symptoms** | **Own maltreatment experience** | **Negative impact on career** | **Quality of life** | **Burden of the pandemic** | **Coping with the pandemic** |
| --- | --- | --- | --- | --- | --- | --- | --- | --- |
| **Age** | 1 | - | - | - | - | - | - | - |
| **Income** | -.05* (.01) n=2470 | 1 | - | - | - | - | - | - |
| **Mental health symptoms** | .17*** (<.001) n=2514 | -.13*** (<.001) N=2469 | 1 | - | - | - | - | - |
| **Own maltreatment experience** | .03 (.09) n=2515 | -.09*** (<.001) N=2470 | .33*** (<.001) N=2514 | 1 | - | - | - | - |
| **Negative impact on career** | -.17*** (<.001) n=2461 | 0 (.98) N=2417 | .16*** (<.001) N=2461 | .14*** (<.001) N=2461 | 1 | - | - | - |
| **Quality of life** | -.24*** (<.001) n=2505 | .22*** (<.001) N=2460 | -.60*** (<.001) N=2504 | -.29*** (<.001) N=2505 | -.18*** (<.001) N=2452 | 1 | - | - |
| **Burden of the pandemic** | .05* (.02) N=2502 | -.08 (<.001) N=2457 | .35*** (<.001) N=2501 | .24*** (<.001) N=2502 | .45*** (<.001) N=2455 | -.40*** (<.001) N=2492 | 1 | - |
| **Coping with the pandemic** | -.09*** (<.001) N=2504 | .13 (<.001) N=2459 | -.38*** (<.001) N=2503 | -.25*** (<.001) N=2504 | -.38*** (<.001) N=2457 | .51*** (<.001) N=2494 | -.60*** (<.001) N=2498 | 1 |

*p-values<.001 are marked with ***, p-values<.01 are marked with ** and p-values<.05 are marked with *.*

**Appendix 3**

*Correlation matrix for the subsample of families.*

| **Variable** | **Age** | **Income** | **Mental health** | **Own maltreat-ment** | **Number of children** | **Age of first child** | **Division of care duties** | **Negative impact career** | **Burden for child** | **Change in parental stress** | **Relation-ship with child** |
| --- | --- | --- | --- | --- | --- | --- | --- | --- | --- | --- | --- |
| **Age** | 1 | - | - | - | - | - | - | - | - | - | - |
| **Income** | .15** (<.01) N=444 | 1 | - | - | - | - | - | - | - | - | - |
| **Mental health symptoms** | .03 (.54) N=453 | -.08 (.11) N=444 | 1 | - | - | - | - | - | - | - | - |
| **Own maltreatment experience** | -.01 (.77) N=453 | -.01 (.77) N=444 | .30 (<.001) N=453 | 1 | - | - | - | - | - | - | - |
| **Number of children** | .23*** (<.001) N=440 | -.11* (.02) N=432 | .02 (.75) N=440 | -.03 (.60) N=440 | 1 | - | - | - | - | - | - |
| **Age of first child** | .67*** (<.001) N=453 | .06 (.20) N=444 | .01 (.83) N=453 | -.01 (.79) N=453 | .36*** (<.001) N=440 | 1 | - | - | - | - | - |
| **Division of care duties** | 0 (.96) N=374 | .05 (.39) N=367 | -.28*** (<.001) N=374 | -.19*** (<.001) N=374 | .05 (.36) N=361 | .02 (.72) N=374 | 1 | - | - | - | - |
| **Negative impact of COVID 19 on career** | -.07 (.14) N=447 | -.08 (.11) N=438 | .21*** (<.001) N=447 | .23*** (<.001) N=447 | -.11* (.02) N=434 | -.06 (.19) N=447 | -.28*** (<.001) N=372 | 1 | - | - | - |
| **Burden of the pandemic for the child** | .06 (.22) N=441 | -.02 (.73) N=432 | .37*** (<.001) N=441 | .25*** (<.001) N=441 | .03 (.48) N=435 | .11* (.02) N=441 | -.30*** (<.001) N=363 | .44*** (<.001) N=435 | 1 | - | - |
| **Change in parental stress** | .10* (<.05) N=431 | .06 (.23) N=423 | -.19*** (<.001) N=431 | -.28*** (<.001) N=431 | .06 (.25) N=425 | .08 (.09) N=431 | .31*** (<.001) N=353 | -.32*** (<.001) N=425 | -.44*** (<.001) N=427 | 1 | - |
| **Relationship with child** | -.01 (.81) N=439 | .09 (.08) N=430 | -.38*** (<.001) N=439 | -.35*** (<.001) N=439 | 0 (.95) N=433 | -.04 (.47) N=439 | .31*** (<.001) N=363 | -.26*** (<.001) N=433 | -.37*** (<.001) N=436 | .34*** (<.001) N=425 | 1 |

*p-values<.001 are marked with ***, p-values<.01 are marked with ** and p-values<.05 are marked with *.*


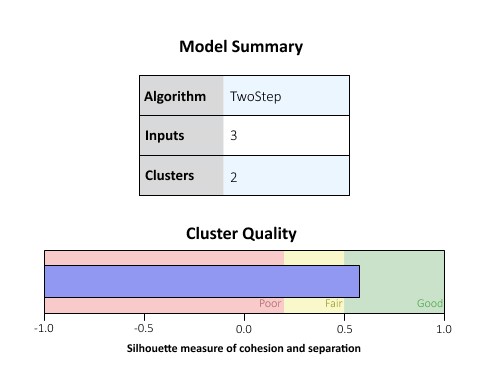


**Appendix 4**

*Results for the cluster quality for the cluster with the whole sample.*


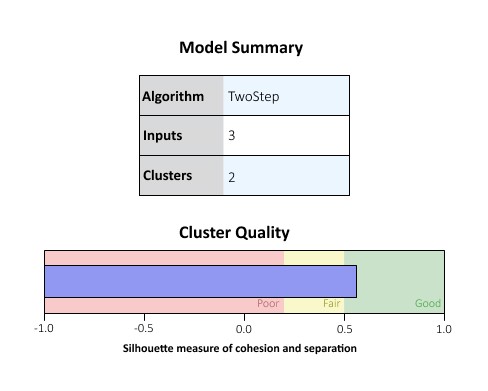


**Appendix 5**

*Results for the cluster quality for the cluster using the subsample of families.*

**Appendix 6**

*Differences between the clusters in the whole sample.*

| **Variable** | **Challenged cluster (N=1298)**  **M (SD)/ n (%)** | **Well-adjusted cluster (N=1190)**  **M (SD)/ n (%)** | **T(df)/ X^2^(df)** | **p** |
| --- | --- | --- | --- | --- |
| **Age** | 51.34 (18.08) | 48.74 (17.89) | 3.59 (2486)*** | <.001 |
| **Income** | 1921.61 (996.15) | 2117.98 (1025.26) | -4.80 (2441)*** | <.001 |
| **Mental health symptoms** | 2.37 (2.45) | 0.76 (1.30) | 20.67 (2002.77)*** | <.001 |
| **Own maltreatment experience** | 0.93 (1.21) | 0.45 (0.86) | 11.42 (2339.91)*** | <.001 |
| **Negative impact of COVID 19 on career** | 3.52 (3.19) | 1.11 (1.95) | 22.71 (2115.23)*** | <.001 |
| **Gender** |  |  | 8.56 (1)** | <.01 |
| female | 705 (54.36%) | 577 (48.49%) |  |  |
| male | 592 (45.64%) | 613 (51.51%) |  |  |
| **Pre-existing psychiatric disorder** |  |  | 160.92 (1)*** | <.001 |
| yes | 329 (26.03%) | 79 (6.78%) |  |  |
| no | 935 (73.97%) | 1087 (93.22%) |  |  |
| **Pre-existing somatic disorder** |  |  | 43.78 (1)*** | <.001 |
| yes | 559 (43.27%) | 360 (30.41%) |  |  |
| no | 733 (56.73%) | 824 (69.59%) |  |  |
| **Living alone** |  |  | 1.86 (1) | .17 |
| yes | 531 (40.91%) | 455 (38.24%) |  |  |
| no | 767 (59.09%) | 735 (61.76%) |  |  |
| **Working from home** |  |  | 10.13 (1)** | <.01 |
| yes | 274 (22.15%) | 190 (16.93%) |  |  |
| no | 963 (77.85%) | 932 (83.07%) |  |  |
| **Income loss** |  |  | 98.18 (1)*** | <.001 |
| yes | 347 (27.15%) | 131 (11.23%) |  |  |
| no | 931 (72.85%) | 1035 (88.77%) |  |  |

**Appendix 7**

*Differences between the clusters in the subsample of families.*

| **Variable** | **Challenged cluster (N=210)**  **M (SD)/ n (%)** | **Well-adjusted cluster (N=212)**  **M (SD)/ n (%)** | **T(df)/ X^2^(df)** | **p** |
| --- | --- | --- | --- | --- |
| **Age** | 40.26 (7.90) | 39.85 (8.83) | 0.5 (420) | .62 |
| **Income** | 1781.04 (838.19) | 1912.99 (794.41) | -1.64 (412) | .10 |
| **Mental health symptoms** | 1.98 (2.12) | 0.74 (1.23) | 7.34 (334.58)*** | <.001 |
| **Own maltreatment experience** | 0.94 (1.31) | 0.38 (0.80) | 5.31 (344.90)*** | <.001 |
| **Number of children** | 1.73 (0.73) | 1.74 (0.82) | -.06 (414) | .95 |
| **Age of first child** | 11.90 (5.88) | 11.16 (7.50) | 1.14 (399.21) | .26 |
| **Division of care duties** | 6.39 (2.11) | 7.65 (2.21) | -5.42 (346)*** | <.001 |
| **Negative impact of COVID 19 on career** | 4.21 (3.09) | 1.76 (2.60) | 8.74 (402.31)*** | <.001 |
| **Gender** |  |  | 3.63 (1) | .06 |
| female | 135 (64.29%) | 117 (55.19%) |  |  |
| male | 75 (35.71%) | 95 (44.81%) |  |  |
| **Pre-existing psychiatric disorder** |  |  | 17.96 (1)*** | <.001 |
| yes | 44 (21.67%) | 15 (7.11%) |  |  |
| no | 159 (78.33%) | 196 (92.89%) |  |  |
| **Pre-existing somatic disorder** |  |  | 1.04 (1) | .31 |
| yes | 44 (21.05%) | 36 (17.14%) |  |  |
| no | 165 (78.95%) | 174 (82.86%) |  |  |
| **Working from home** |  |  | 7.39 (1)** | <.01 |
| yes | 61 (29.19%) | 37 (17.87%) |  |  |
| no | 148 (70.81%) | 170 (82.13%) |  |  |
| **Partner working from home** |  |  | 3.17 (1) | .08 |
| yes | 46 (28.75%) | 35 (20.35%) |  |  |
| no | 114 (71.25%) | 137 (79.65%) |  |  |
| **Income loss** |  |  | 28.90 (1)*** | <.001 |
| yes | 82 (40.00%) | 34 (16.27%) |  |  |
| no | 123 (60.00%) | 175 (83.73%) |  |  |

**Appendix 8**

*Results of the moderation analysis in the whole sample. Cluster values are expressed in a logg-odds metric. The lines representing values one SD under the mean for negative impact on career (-1; representing low negative impact of the pandemic on the career), mean negative impact on career (0) and one SD above the mean of negative impact on career (1; representing high negative impat of the pandemic on the career).*

**Appendix 9**

*Results of the moderation analysis in the subsample for families. Cluster values are expressed in a logg-odds metric. The lines representing values one SD under the mean for negative impact on career (-1; representing low negative impact of the pandemic on the career), mean negative impact on career (0) and one SD above the mean of negative impact on career (1; representing high negative impact of the pandemic on the career).*
